# Impaired T cell-mediated hepatitis in peroxisome proliferator activated receptor alpha (PPARα)-deficient mice

**DOI:** 10.1186/s40659-018-0153-z

**Published:** 2018-02-15

**Authors:** Ian N. Hines, Michael Kremer, Sherri M. Moore, Michael D. Wheeler

**Affiliations:** 1 Department of Nutrition Science, College of Allied Health Sciences, East Carolina University, Health Sciences Bldg. Room 4165F, Greenville, NC 27834 USA; 2 Department of General Surgery, University of Ulm, Ulm, Germany; 3 Department of Medicine, University of North Carolina, Chapel Hill, NC 27599 USA

**Keywords:** Inflammation, Cytokines, T helper phenotype, Interferon gamma

## Abstract

**Background:**

Peroxisome proliferator activated receptor alpha (PPARα), a regulator of enzymes involved in β oxidation, has been reported to influence lymphocyte activation. The purpose of this study was to determine whether PPARα plays a role in T cell-mediated hepatitis induced by Concanavalin A (ConA).

**Methods:**

Wild type (wt) or PPARα-deficient (PPARα^−/−^) mice were treated with ConA (15 mg/kg) by intravenous injection 0, 10 or 24 h prior to sacrifice and serum and tissue collection for analysis of tissue injury, cytokine response, T cell activation and characterization.

**Results:**

Ten and 24 h following ConA administration, wt mice had significant liver injury as demonstrated by serum transaminase levels, inflammatory cell infiltrate, hepatocyte apoptosis, and expression of several cytokines including interleukin 4 (IL4) and interferon gamma (IFNγ). In contrast, PPARα^−/−^ mice were protected from ConA-induced liver injury with significant reductions in serum enzyme release, greatly reduced inflammatory cell infiltrate, hepatocellular apoptosis, and IFNγ expression, despite having similar levels of hepatic T cell activation and IL4 expression. This resistance to liver injury was correlated with reduced numbers of hepatic natural killer T (NKT) cells and their in vivo responsiveness to alpha-galactosylceramide. Interestingly, adoptive transfer of either wt or PPARα^−/−^ splenocytes reconstituted ConA liver injury and cytokine production in lymphocyte-deficient, severe combined immunodeficient mice implicating PPARα within the liver, possibly through support of IL15 expression and/or suppression of IL12 production and not the lymphocyte as the key regulator of T cell activity and ConA-induced liver injury.

**Conclusion:**

Taken together, these data suggest that PPARα within the liver plays an important role in ConA-mediated liver injury through regulation of NKT cell recruitment and/or survival.

## Background

Growing experimental and clinical data highlight a complex interaction among lipids, immune cells, and the hepatic inflammatory responses [1–4]. Accumulation of lipid leads to inflammatory cell infiltration and activation which promotes secondary tissue injury and organ dysfunction [1]. Key aspects in the regulation of this process remain unclear, particularly the intersection of lipid metabolism and immune cell function whether it be direct or indirect through hepatocellular stress/damage. Peroxisome proliferator activated receptor alpha (PPARα) is a nuclear hormone receptor associated with proliferation of peroxisomes in the hepatocytes of rodents in response to a number of naturally occurring as well as synthetic compounds [5]. PPARα is also a regulator of the production of a number of enzymes including acyl Coenzyme A oxidase (AOX) involved in the metabolism of fatty acids within the liver [6, 7]. As a result, mice deficient in this AOX present with an age dependent increase in the accumulation of hepatocellular fat or steatosis.

PPARα also plays a prominent role in inflammatory response [8–10]. For example, foam cell formation is reduced by the ligand-specific activation of PPARα in a model of hypercholesterolemia induced atherosclerosis [11]. Human monocyte-derived macrophages have also shown sensitivity to PPARα ligand activation with increased levels of apoptosis [9, 10, 12]. Further investigation has revealed an inhibitory effect of PPARα on the pro-inflammatory transcription factor nuclear factor kappa B (NFκB), a possible mechanism for its anti-inflammatory actions [13]. Jones et al. also report the presence of PPARα in CD4^+^ T lymphocytes in rodents [14]. As with macrophages, PPARα in T lymphocytes appears to regulate the activity of NFκB suggesting a common mechanism and role in immune cell function [14]. Interestingly, studies have also demonstrated a dysregulation of cytokine production in T lymphocytes from PPARα-deficient (PPARα^−/−^) mice whereby deficient cells produce significantly larger quantities of interferon gamma (IFNγ) in response to anti-CD3/anti-CD28 activation [15]. Such data would suggest that PPARα is capable of modulating the function and immunological response of a variety of immune cells from macrophages to T cells and therefore may play a significant role in the determination of T cell responsiveness in vivo.

Concanavalin A (ConA) is a plant lectin capable of inducing severe T cell mediated hepatitis in the mouse [16]. ConA activates CD1d-dependent intrahepatic natural killer T (NKT) cells to produce a number of pro-inflammatory mediators including tumor necrosis factor alpha, interleukin 4 (IL4), and IFNγ [17–19]. Given the presence of PPARα in T cells, its apparent regulation of T cell and macrophage activation, and its influence on hepatocellular lipid metabolism, PPARα lies at the unique nexus of lipid metabolism and immunological function. The current study was thus aimed at understanding the impact of PPARα in the complex setting of T cell-mediated hepatitis. To this end, we have administered ConA to wild type and PPARα^−/−^ mice and revealed a surprising and profoundly protective effect of PPARα deficiency on ConA-mediated, T cell-dependent liver injury, a protection likely related to reductions in hepatic NKT cell number and function.

## Methods

### Animals

Eight to twelve week old male C57Bl/6 mice, PPARα-deficient (PPARα^−/−^) mice [20], or severe combined immunodeficient (SCID) mice on a C57Bl/6 background were purchased from Jackson Laboratories (Bar Harbor, ME). All animals were housed in specific pathogen free conditions with 12 h light/dark cycles and free access to food and water. All subsequent procedures described were approved by the Institutional Animal Care and Use Committee at the University of North Carolina at Chapel Hill and complied with the “Guide for the Care and Use of Laboratory Animals”.

### Lipopolysaccharide treatment

Male mice, either wild type or PPARα^−/−^, were administered lipopolysaccharide (LPS; 1 mg/kg, Sigma, St. Louis, MO) by intraperitoneal injection in 200 μl of normal saline or saline alone as control 6 h prior to sacrifice.

### Concanavalin A (ConA) mediated hepatitis

Male mice, either wild type or PPARα^−/−^, were administered Concanavalin (ConA; Sigma, St. Louis, MO) at a dose of 15 mg/kg in sterile saline via tail vein injection as has previously been described [21]. Mice were then anesthetized with ketamine and xylazine (100 and 10 mg/kg respectively) 10 or 24 h following injection, the diaphragm severed to effect euthanasia, and serum and tissue collected.

### α-Galactosylceramide (αGal) treatment

Male mice, either wild type or PPARα^−/−^, were administered αGal (Funakoshi, Tokyo, Japan) by intravenous injection at a dose of 10 μg/mouse through the tail vein as previously reported [2]. Mice were then euthanized 12 h as described above to assess liver injury and cytokine production.

### Liver enzyme assessment

Blood was collected from the inferior vena cava from anesthetized mice 10 h following ConA administration into sterile microcentrifuge tubes. Blood was allowed to clot on ice for a period of 10 min after which it was centrifuged at 12,000×*g* allowing for collection of serum. Serum levels of alanine aminotransferase (ALT) and aspartate aminotransferase (AST) were measured by the Clinical Chemistry Laboratory at the University of North Carolina at Chapel Hill using standard techniques.

### Histopathology and immunohistochemistry

Liver tissue was collected at the time of sacrifice and placed in 10% buffered formalin (Thermo-Fisher Scientific, Waltham, MA) at 4 °C for 24 h. After fixation, the tissue was embedded in paraffin and 7 μm thick sections cut. Sections were then deparaffinized, rehydrated, and stained with hematoxylin and eosin. Additionally, some sections were stained for the T cell marker, CD3ε (Thermo-Fisher Scientific), as previously described [22]. Sections were examined under routine light microscopy at 100× and 400× magnification and images captured using an Olympus DP70 digital camera.

### Terminal UTP nick end labeling (TUNEL) staining

To assess liver cell death, deparaffinized sections were stained for DNA fragmentation using a commercially available kit (In situ cell death detection kit, Roche, Indianapolis, IN, Cat# 11684795910) according to the manufacturer’s recommendations as previously described [21]. Stained sections were viewed by fluorescent microscopy and images capture with an Olympus DP70 digital camera. Five random high powered fields were observed and positive cells counted.

### Hepatic triglyceride quantification

Liver triglycerides were quantified using kit from Sigma (Triglyceride Reagent, Cat.# T2449, St. Louis MO) according to the manufacturer’s recommendations as previously described by our group [2]. Triglyceride content was normalized to wet weight of tissue used in the assay.

### Real time polymerase chain reaction

Total RNA (5 μg) isolated with Trizol reagent (Thermo-Fisher) was reverse transcribed using a kit obtained from Applied Biosystems (High Capacity Reverse Transcription Kit Cat.# 4368814, Foster City, CA). For quantification of message expression, 250 ng of cDNA was amplified in a Eppendorf RealPlex^2^ using the primers listed in Table 1 (except IL15 where primers were purchased from Real Time Primers, Elkins Park, PA) in the presence of Sybr Green I (Maxima Sybr Green Reagent, Cat.# K0221, Applied Biosystems) using 45 cycles of a three step protocol, 95 °C for 10 s, 57 °C for 15 s, and 72 °C for 20 s. All message expression was normalized to the housekeeping gene β actin and expressed as gene expression relative to the wild type 0 h animals using the comparative ct method. Amplification of a single product was verified by analysis of post-amplification product dissociation temperatures (i.e. melt curves).

**Table 1:** Primer sequences used for quantitative PCR analysis

Gene	Primer sequence
T-bet	For 5ʹ-TGCCCGAACTACAGTCACGAAC-3ʹ
	Rev 5ʹ-AGTGACCTCGCCTGGTGAAATG-3ʹ
Tumor necrosis factor alpha (TNFα)	For 5ʹ-AGCCCACGTAGCAAACCACCAA-3ʹ
	Rev 5ʹ-ACACCCATTCCCTTCACAGAGCAAT-3ʹ
Interferon γ (IFNγ)	For 5ʹ-TCAAGTGGCATAGATGTGGAAGAA-3ʹ
	Rev 5ʹ-TGGCTCTGCAGGATTTTCATG-3ʹ
Interleukin 12 p40 (IL12p40)	For 5ʹ-GGAAGCACGGCAGCAGAATA-3ʹ
	Rev 5ʹ-AACTTGAGGGAGAAGTAGGAATGG-3ʹ
Interleukin 4 (IL4)	For 5ʹ-ACAGGAGAAGGGACGCCAT-3ʹ
	Rev 5ʹ-GAAGCCCTACAGACGAGCTCA-3ʹ
Interleukin 5 (IL5)	For 5ʹ-AGCACAGTGGTGAAAGAGACCTT-3ʹ
	Rev 5ʹ-TCCAATGCATAGCTGGTGATTT-3ʹ
Interleukin 10 (IL10)	For 5ʹ-GGTTGCCAAGCCTTATCGGA-3ʹ
	Rev 5ʹ-ACCTGCTCCACTGCCTTGCT-3ʹ
Acyl-CoA oxidase (AOX)	For 5ʹ-CTTGTTCGCGCAAGTGAGG-3ʹ
	Rev 5ʹ-CAGGATCCGACTGTTTACC-3ʹ
Cluster of differentiation 1d (CD1d)	For 5ʹ-TCCTAGAGGCAGGGAAGTCA-3ʹ
	Rev 5ʹ-AGCATTTGGCAGGAAATCAC-3ʹ
Peroxisome proliferator activated receptor alpha (PPARα)	For 5ʹ-GTGGCTGCTATAATTTGCTGTG-3ʹ
	Rev 5ʹ-GAAGGTGTCATCTGGATGGGT-3ʹʹ
Liver fatty acid binding protein (LFABP)	For 5ʹ-GTGGTCCGCAATGAGTTCAC-3ʹ
	Rev 5ʹ-GTATTGGTGATTGTGTCTCC-3ʹ
β actin	For 5ʹ-AGGTGTGCACCTTTTATTGGTCTCAA-3ʹ
	Rev 5ʹ-TGTAGTAAGGTTTGGTCTCCCT-3ʹ

### Flow cytometry

Liver mononuclear cells and total splenocytes were obtained as described previously [2, 21]. Isolated cells were stained for the immune cell markers T cell receptor beta (TCRβ; BD Pharmingen, San Jose, CA), CD4 (Thermo-Fisher), pan natural killer cell (DX5; Thermo-Fisher), and the activation marker, CD69 (Thermo-Fisher) at a 1:100 dilution for 30 min at room temperature. For spleen cells, whole spleens were homogenized between glass slides centrifuged at 500×*g*, and filtered through a 30 μm sterile filter followed by staining with the above listed antibodies. Again, cells were stained with the above listed antibodies. Cells were then analyzed and relative numbers expressed by % of total mononuclear cells and/or % of total liver TCRβ^+^ cells in liver mononuclear cell fraction.

### Enzyme-linked immunosorbent assay

Serum and/or tissue culture media IL12, IFNγ, or IL4 protein was determined using a kit from R&D systems (IL12, Cat#M1270; IFNγ, Cat#MIF00; and IL4, Cat#M4000B) per manufacturer’s instruction as previously described [21]. Samples were compared to a standard curve and values expressed per mg of liver protein.

### In vitro ConA activation

Wild type or PPARα^−/−^ mononuclear cells were isolated as described previously. For activation studies, 1 × 10^5^ liver mononuclear cells or spleen cells were incubated in a 96 well plate in 300 μl of RPMI media (Invitrogen) in the presence or absence of 1 μg/ml ConA (Sigma) for 72 h at 37 °C and 5% CO_2_. Following incubation, media was collected and assessed for IFNγ and IL4 protein by ELISA as described above.

### SCID lymphocyte reconstitution

Total splenocytes (2 × 10^7^) were isolated as described above from wild type and PPARα^−/−^ mice. Red blood cells were removed by incubation in red blood cell lysis solution for 10 min at room temperature. Cell viability and number were assessed by trypan blue exclusion. Splenocytes (2 × 10^7^) were resuspended in 100 μl of PBS and injected intravenously into SCID recipients through the tail vein. SCID mice administered PBS alone served as controls for these experiments. Seven days following reconstitution, animals were administered ConA (15 mg/kg). Ten hours later, serum and tissue were collected to assess T cell reconstitution, liver injury, and cytokine expression.

### Statistical analysis

Data are presented as mean ± standard error of the mean (SEM) of 4 or more animals per group. Data were analyzed using the non-parametric Mann–Whitney Rank Sum Test or analysis of variance where significance was set at p < 0.05.

## Results

### Characterization of PPARα^−/−^ mice

**Fig. 1:**
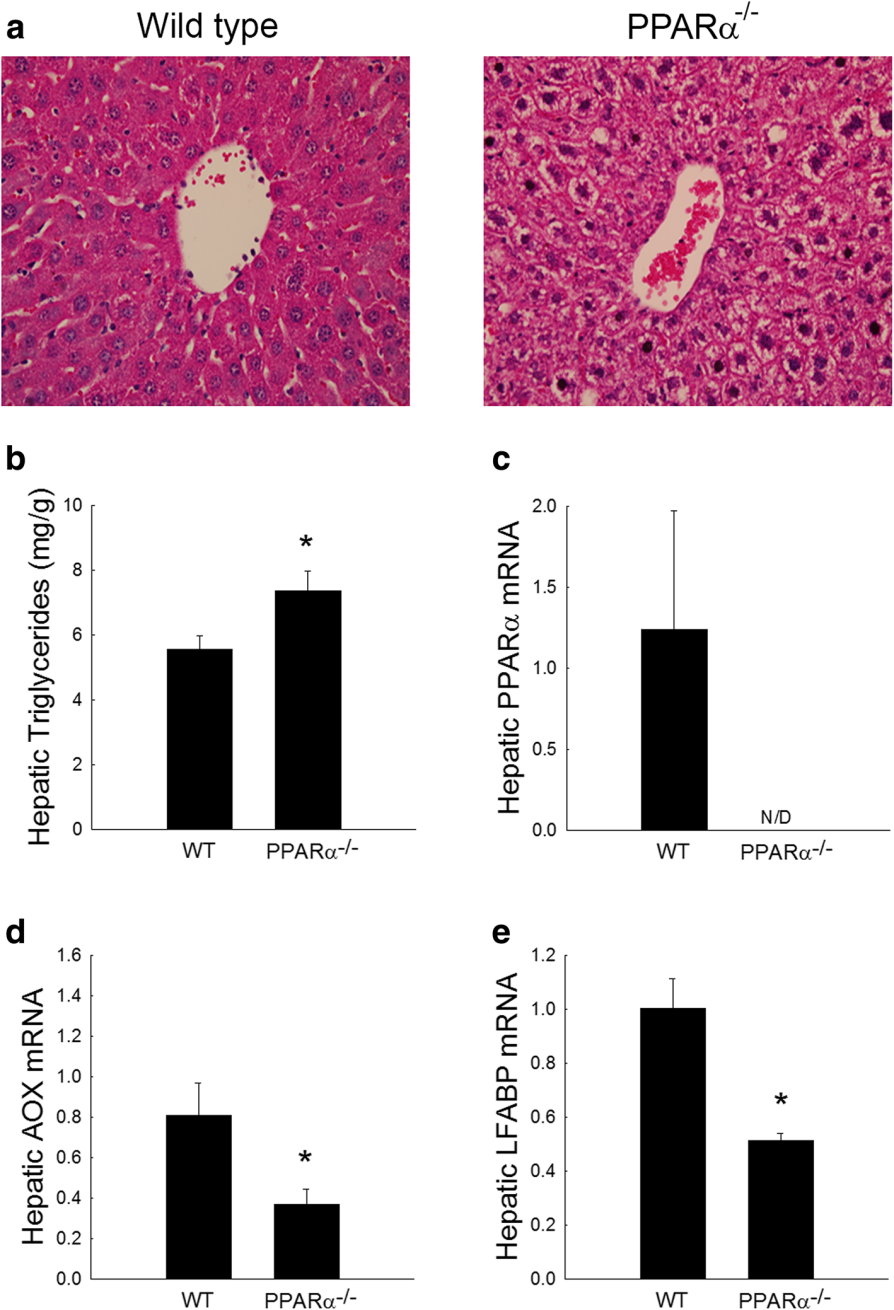
Characterization of PPARα deficient livers. **a** Hematoxylin and Eosin stained liver sections from untreated wild type and PPARα^−/−^ deficient mice. Representative ×400 photomicrographs presented. **b** Hepatic triglyceride content in control, untreated wild type and PPARα^−/−^ mice. **c** Hepatic mRNA expression for PPARα (**c**), acyl CoA oxidase (**d**; AOX) and liver fatty acid binding protein (**e**; LFAPB) in untreated wild type and PPARα^−/−^ mice. *p < 0.05 vs. wild type control. n = 6 animals per group. *N/D* not detected

PPARα is a known regulator of lipid metabolism with significant relevance to liver function [6, 23]. Figure 1 characterizes the impact of a loss of PPARα on hepatic lipid accumulation as well as lipid metabolizing/transporting gene expression. Ten week old PPARα deficient mice have increased microvesicular lipid accumulation as assessed by routine histopathology (Fig. 1a) and a significant increase in triglyceride content (Fig. 1b). Expression analysis confirms absence of PPARα in our knockout mice (Fig. 1c) and this loss correlates with reduced hepatic acyl-CoA oxidase (Fig. 1d) and liver fatty acid binding protein (Fig. 1e) expression as has previously been reported [6]. Together, these data are consistent with previous reports and highlight the impact of a loss of PPARα on the hepatic microenvironment and provide a platform to study its impact on ConA-induced, T cell-mediated tissue injury.

### Deficiency in PPARα inhibits Concanavalin A (ConA)-mediated hepatitis

**Fig. 2:**
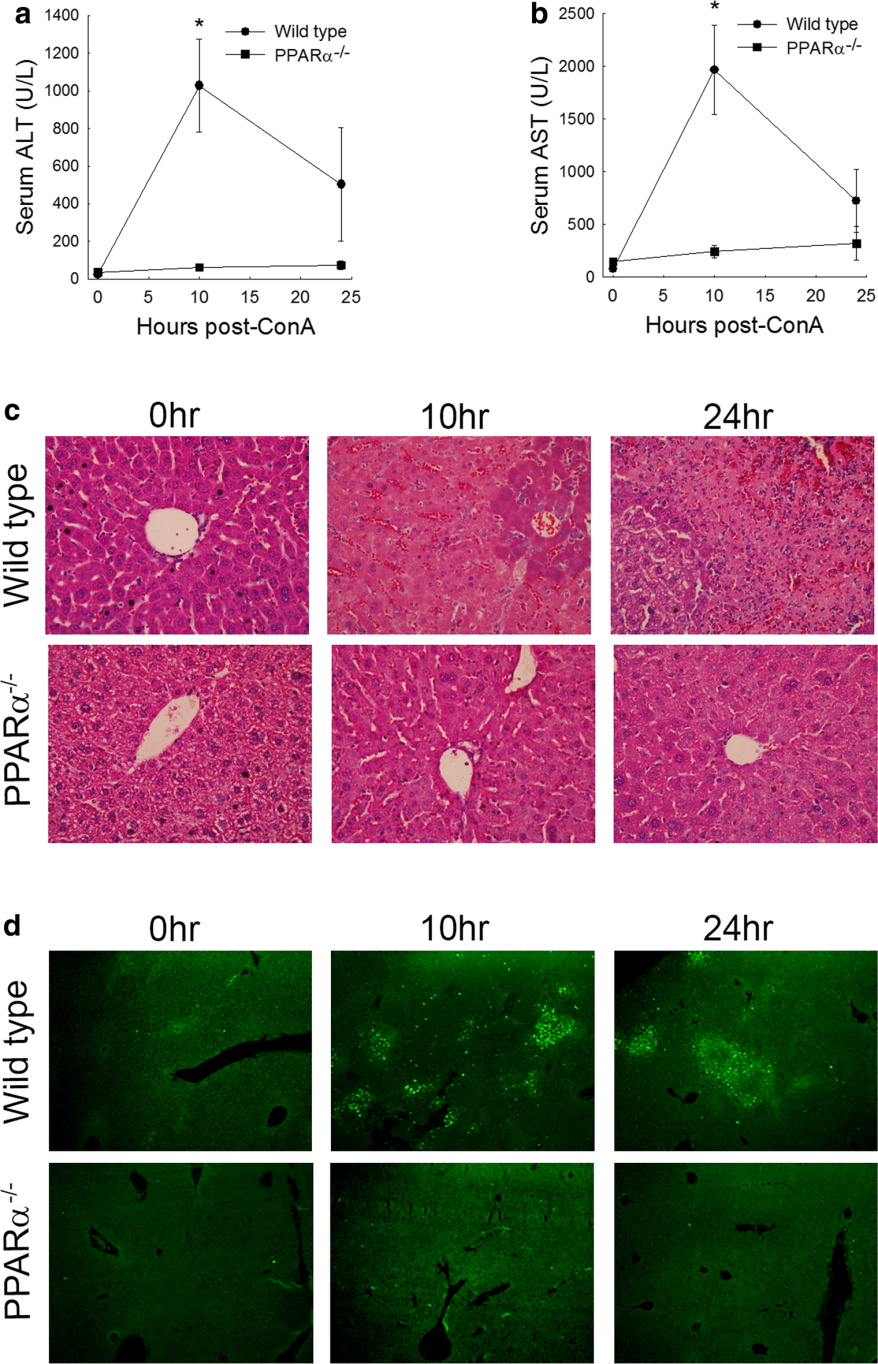
Serum enzyme levels and histopathology from wild type and PPARα^−/−^ deficient mice administered ConA at 15 mg/kg. **a** Serum alanine aminotransferase (ALT) levels 0, 10 or 24 h following ConA administration in wild type and PPARα^−/−^ mice. **b** Serum aspartate aminotransferase (AST) levels 0, 10, and 24 h following ConA administration in wild type and PPARα^−/−^ mice. **c** Hematoxylin and eosin stained liver sections from wild type and PPARα^−/−^ mice 0, 10, and 24 h following ConA administration. Representative ×400 photomicrographs are shown. **d** Terminal UTP nick-end labeling (TUNEL) staining of liver sections from wild type and PPARα^−/−^ mice 0, 10, and 24 h following ConA administration. Representative ×100 photomicrographs are shown *p < 0.05 vs 0 h wild type value. ^+^p < 0.05 vs. wild type at 10 h post-injection. n = 4 animals per group

ConA administration is an established model of T cell-mediated hepatitis in rodents [16–19, 24]. Doses from 10 to 20 mg/kg body weight are associated with significant NKT cell-dependent hepatocellular injury [16, 21]. To determine the role that PPARα plays in ConA-mediated, T cell dependent liver injury, 10 week old wild type and PPARα^−/−^ mice were given 15 mg/kg ConA by intravenous injection. Ten hours following this dose of ConA, serum ALT and AST levels were significantly elevated in wild type mice (Fig. 2a, b) with levels remaining elevated through 24 h post-injection. This increase in serum levels of ALT or AST was not observed in PPARα^−/−^ mice 10 h post-injection (Fig. 2a, b). Consistent with serum measurements of liver injury, histopathological assessment of livers from ConA pre-treated wild type mice revealed large areas of necrosis with the appearance of inflammatory cell infiltrate (Fig. 2c). Examination of liver sections from PPARα^−/−^ mice treated with ConA confirmed the protective effect of this deficiency.

ConA has also been shown to induce liver injury through the induction of hepatocellular apoptosis via a Fas-dependent mechanism [25–27]. To determine if the ConA-induced apoptotic cell death was also disrupted in PPARα^−/−^ mice, liver sections from wild type and PPARα^−/−^ mice were subjected to the TUNEL assay to assess DNA fragmentation, a marker of apoptotic cell death. Consistent with serum enzyme measures and histopathological signs of liver damage, wild type mice given ConA had increased numbers of TUNEL positive cell number when compared to their untreated controls at 10 and 24 h post-injection (Fig. 2d). In contrast, PPARα^−/−^ livers were resistant to ConA-induced increases in hepatocellular apoptosis, a finding consistent with an absence of liver injury. Taken together, these data suggest that PPARα may be involved in the early development of ConA-induced, T cell mediated, liver injury in mice.

### Splenic and hepatic T cells are activated in wild type and PPARα^−/−^ mice in response to ConA

**Fig. 3:**
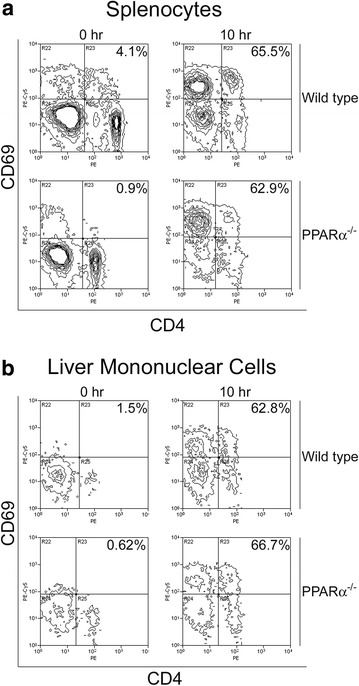
Flow cytometric evaluation of CD4^+^ T lymphocyte activation at 0 and 10 h post-ConA injection using the early activation marker CD69. Total splenocytes (**a**) or liver mononuclear cells (**b**) stained for CD4 (x axis) and CD69 (y axis). Percentages shown are of CD4^+^ cells positive or negative for CD69 with representative contour plots from 4 mice per group shown

ConA is known to activate both peripheral and intrahepatic T cells [18, 19]. More specifically, the activation of intrahepatic CD4^+^ natural killer T (NKT) cells is a key component of ConA-induced liver injury [17]. Splenic and intrahepatic T cells from wild type and PPARα^−/−^ mice were therefore isolated and stained for the T cell marker CD4 in combination with the early activation marker, CD69 [28]. As shown in Fig. 3, wild type and PPARα^−/−^ splenic and intrahepatic CD4^+^ T cells were activated to similar levels 10 h following ConA administration. These data confirm that T cells from wild type and PPARα^−/−^ mice respond similarly to ConA exposure.

### Deficiency in PPARα alters ConA-induced cytokine expression in liver

**Fig. 4:**
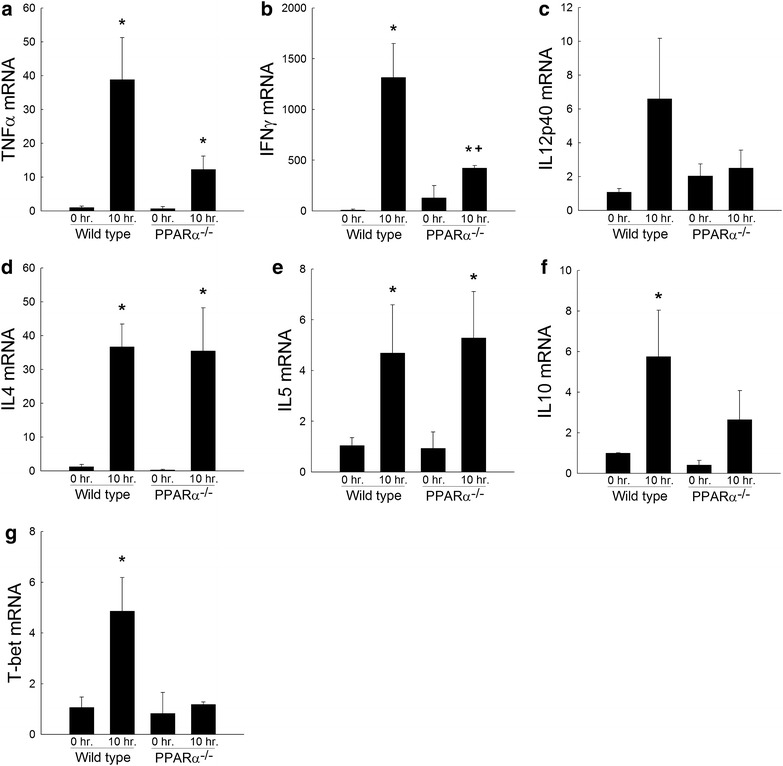
Cytokine expression from total liver RNA as assessed by real-time PCR in wild type and PPARα^−/−^ mice 0 or 10 h following a single dose of ConA. **a** Tumor necrosis factor alpha (TNFα), **b** interferon gamma (IFNγ), and **c** interleukin 12p40; **d** interleukin 4, **e** interleukin 5 (IL5), **f** interleukin 10 (IL10), and **g** T-bet. *p < 0.05 vs. wild type at 0 h. ^+^p < 0.05 vs. wild type at 10 h post-injection. n = 4 animals per group

A number of studies have demonstrated the ability of ConA to induce pro-inflammatory cytokine expression in the liver and the importance of this cytokine production for the development of hepatocellular injury [18, 19, 24, 29, 30]. Indeed, deletion of interleukin 4 (IL4) or interferon gamma (IFNγ) has been associated with substantial reductions in ConA-induced liver injury [18, 19]. Given the importance of cytokines to the development of ConA-induced hepatitis, it was hypothesized that the cytokine response would be impaired in PPARα^−/−^ mice when compared to their wild type controls. As shown in Fig. 4, wild type mice given ConA present with significant increases in a number of inflammatory mediators associated with acute hepatitis including tumor necrosis factor alpha (TNFγ; Fig. 4a), certain T_h_1 type cytokines including interferon gamma (IFNγ; Fig. 4b), and interleukin 12 (IL12; Fig. 4c) and certain Th_2_ type cytokines including interleukin 4 (IL4; Fig. 4d) interleukin 5 (IL5; Fig. 4e), and interleukin 10 (IL10; Fig. 4f). PPARα^−/−^ mice administered the same dose of ConA had reduced expression of key T_h_1 type cytokines involved in ConA mediated liver injury, specifically IFNγ but similar levels of T_h_2 type cytokines such as IL4 and IL5 when compared to their ConA-treated wild type controls. These data suggest that PPARα is involved, either directly or indirectly, in the activation of the T_h_1-dependent, IFNγ mediated, inflammatory response caused by ConA administration.

It is becoming increasingly apparent that certain transcription factors play important roles in the differentiation of T cells towards T_h_1 or T_h_2 phenotypes [31–33]. T-bet, a T box transcription factor primarily expressed in T cells, is associated with the expression of T_h_1-type cytokines including IFNγ [34]. Furthermore, activation of T-bet has been shown to be crucial to the development of ConA-mediated hepatitis [35]. Given the reduction in expression of IFNγ in PPARα^−/−^ mice following ConA when compared to ConA-treated wild type controls, we tested the hypothesis that PPARα positively regulates expression of this T_h_1-associated transcription factor. T-bet expression is strongly up-regulated in the livers of wild type mice 10 h following ConA administration (Fig. 4g). In contrast, deficiency in PPARα prevents the up-regulation of this T_h_1-associated transcription factor in the liver (Fig. 4g). Together, these data, in conjunction with the reductions in cytokine expression, suggest that PPARα does indeed play a role, either directly or indirectly, in the activation of the T_h_1-associated transcription factor T-bet following ConA administration.

**Fig. 5:**
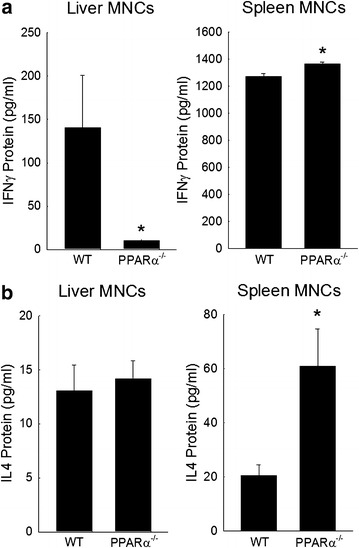
In vitro IFNγ and IL4 protein production in wild type and PPARα^−/−^ mice. Wild type or PPARα^−/−^ splenic or liver mononuclear cells (MNCs) were isolated from untreated animals and exposed to ConA (1 μg/ml) for 72 h). Protein expression for IFNγ (**a**) and IL4 (**b**) were then measured in culture media by ELISA. *p < 0.05 vs. ConA-treated wild type cells. n = 4 experiments

To better understand the defects that are associated with a deficiency in PPARα, wild type and PPARα-deficient liver and spleen mononuclear cells (MNCs) were isolated from untreated animals and cultured in the presence or absence of ConA (1 μg/ml) for 72 h. Media was then analyzed for the presence of IFNγ and IL4 by ELISA. As shown in Fig. 5, splenocytes and hepatic MNCs responded to ConA stimulation with the production of large quantities of both IFNγ and IL4. Absence of PPARα led to a significant reduction in IFNγ production by hepatic MNCs. Interestingly, production of IL4 by these hepatic MNCs was not affected by absence of PPARα. Moreover, splenic MNCs from PPARα deficient mice showed significant increases in both IFNγ and IL4 production when compared to similarly treated wild type MNCs. Together, these in vitro data further confirm the selective impairment of liver derived mononuclear cell production of IFNγ.

### PPARα^−/−^ mice have reduced numbers of liver NKT cells

**Fig. 6:**
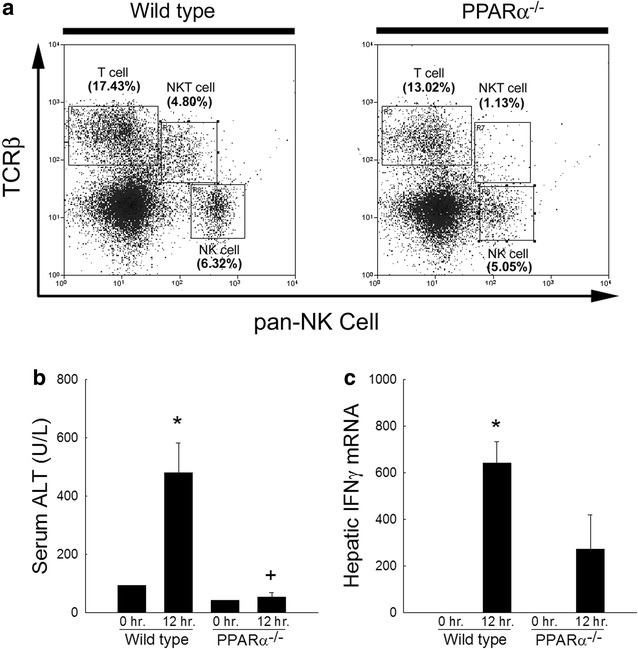
Evaluation of hepatic NKT cells in wild type and PPARα^−/−^ mice. **a** Scatter plots of liver mononuclear cells stained for TCRβ (y axis) and NK cell markers (DX5 and NK1.1, x axis) from untreated wild type or PPARα^−/−^ mice. Representative scatter plots from 4 individual mice in each group are shown. Percentages represent those from total isolated liver mononuclear cells. **b** Serum alanine aminotransferase levels 0 or 12 h following administration of alpha-galactosylceramide (αGal) in wild type and PPARα^−/−^ mice. **c** Hepatic interferon gamma (IFNγ) gene expression 0 or 12 h following αGal administration in wild type and PPARα^−/−^ mice. *p < 0.05 vs. wild type at 0 h. ^+^p < 0.05 vs. wild type at 10 h post-injection. n = 3–4 animals per group

ConA-mediated liver injury requires CD1d-dependent NKT cell activation [17]. Given the profound protection against ConA-induced liver injury associated with a deficiency in PPARα, we examined the NKT cell populations in untreated wild type and PPARα^−/−^ mice. As shown in Fig. 6a, untreated wild type mice have significant numbers of TCRβ and pan-NK positive cells, NKT cells (4.8% of total liver mononuclear cells, 21.5% of hepatic TCRβ^+^ lymphocytes) in the liver. In contrast, PPARα^−/−^ mice have significantly reduced numbers of NKT cells (1.13% of liver mononuclear cells, 7.9% of hepatic TCRβ^+^ lymphocytes) within the liver despite having similar levels of TCRβ positive and pan-NK negative cells (T cells) and TCRβ negative and pan-NK positive cells (NK cells). Taken together, these data implicate PPARα in the development, recruitment, or differentiation of hepatic NKT cells. Furthermore, these data provide a mechanism by which PPARα may regulate ConA-induced T cell-mediated hepatitis.

To further evaluate the functionality of NKT cells directly, wild type mice or PPARα^−/−^ mice were administered alpha galactosylceramide (αGal), a potent and specific activator of NKT cells [36]. Twelve hours following injection, mice were sacrificed and serum and tissue collected for liver enzyme release and pro-inflammatory cytokine production. As shown in Fig. 6b, absence of PPARα resulted in reduced αGal-induced liver injury as assessed by serum ALT levels as well as a reduction in IFNγ gene expression (Fig. 6c) following αGal administration when compared to similarly treated wild type mice. These data further confirm the dysfunction of hepatic NKT cells within PPARα^−/−^ mice.

### PPARα^−/−^ splenocytes are capable of restoring ConA-dependent liver injury in SCID mice

**Fig. 7:**
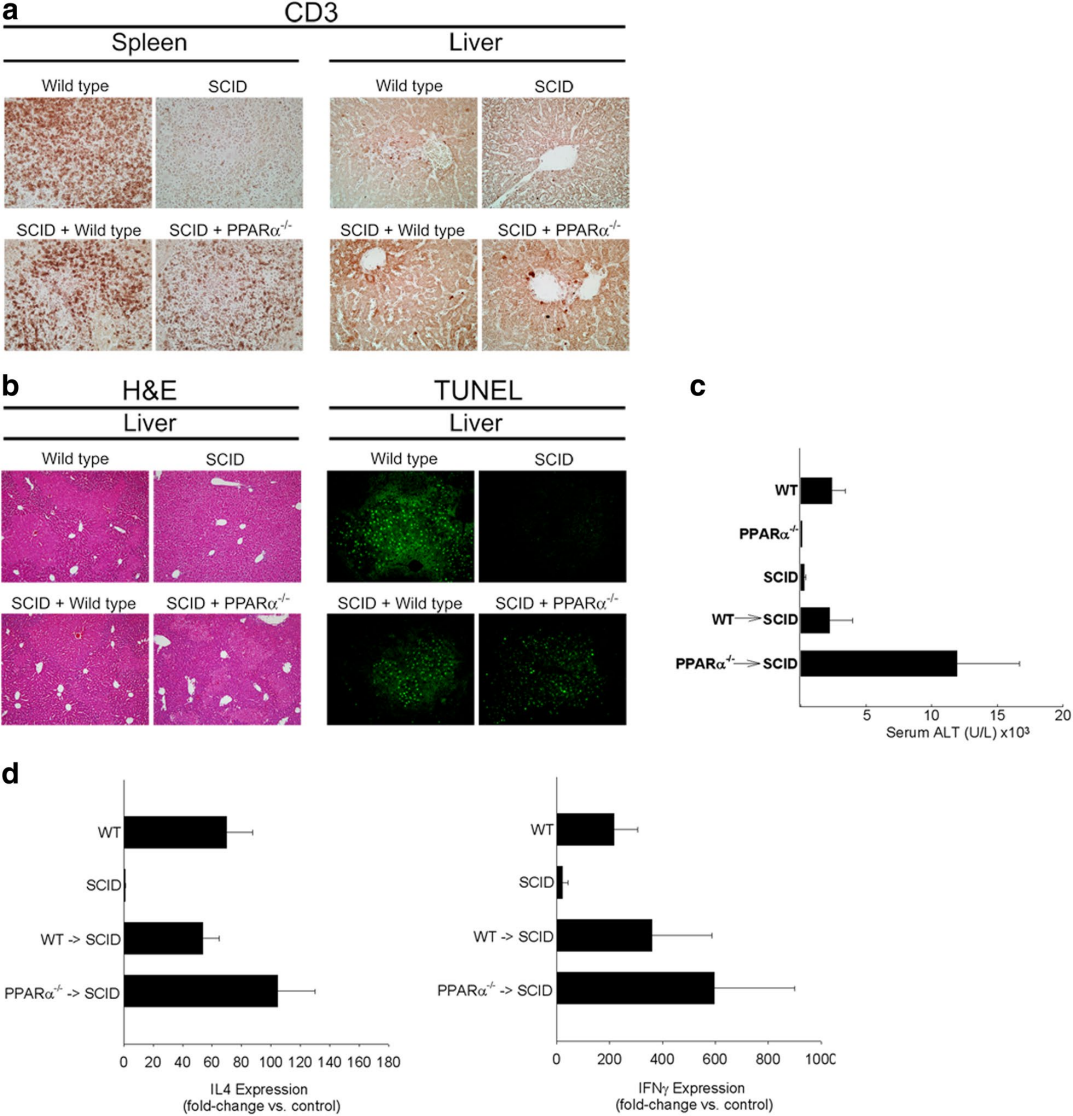
Reconstitution of severe combined immunodeficient (SCID) mice with wild type or PPARα^−/−^ splenocytes reconstitutes ConA mediated liver injury. **a** Immunohistochemical assessment of CD3^+^ cells within the spleens and livers of wild type and SCID mice as well as SCID mice reconstituted with either wild type or PPARα^−/−^ splenocytes. **b** Hematoxylin and Eosin stained or terminal UTP nick end labeled (TUNEL) stained sections from wild type and SCID mice as well as SCID mice reconstituted with either wild type or PPARα^−/−^ splenocytes 10 h following treatment with ConA (15 mg/kg). Representative ×100 photomicrographs presented. **c** Serum alanine aminotransferase (ALT) levels in wild type or SCID mice or SCID mice reconstituted with either wild type or PPARα^−/−^ splenocytes 10 h after intravenous treatment with 15 mg/kg ConA. **d** IL4 and IFNγ message expression in wild type or SCID mice or SCID mice reconstituted with either wild type or PPARα^−/−^ splenocytes 10 h after intravenous treatment with 15 mg/kg ConA. n = 4 animals per group

To determine if the reductions in NKT cell number was indeed due to the absence of PPARα within other cell populations and not to an absence of this transcription factor in NKT cells directly, SCID mice, which express normal levels of PPARα within the liver parenchymal and non-parenchymal cells, were reconstituted with total wild type or PPARα^−/−^ splenocytes. Seven days following lymphocyte reconstitution, wild type mice, SCID mice, and SCID mice reconstituted with either wild type or PPARα^−/−^ splenocytes were administered ConA (15 mg/kg). Reconstitution was verified by immunohistochemical detection of CD3ε in the spleen and liver. As shown in Fig. 7a, SCID mice have no CD3ε positive cells within the spleen or liver whereas SCID mice reconstituted with wild type or PPARα^−/−^ splenocytes showed splenic and hepatic repopulation of CD3ε positive cells to a comparable to that of untreated wild type mice. Ten hours following ConA administration, wild type mice showed significant hepatocellular injury (Fig. 7b) while PBS-treated SCID mice were completely resistant to ConA liver injury as assessed by routine histopathology and TUNEL staining as has previously been reported [21, 37]. Adoptive transfer of wild type splenocytes to SCID mice restored ConA-induced liver injury as assessed by histopathology and TUNEL staining (Fig. 7b) and serum transaminase levels (Fig. 7c). Interestingly, SCID mice reconstituted with PPARα^−/−^ splenocytes showed substantial enhancements in serum ALT when compared to wild type mice reconstituted SCID mice given ConA (Fig. 7c). Consistent with the restoration of ConA-induced liver injury, SCID mice reconstituted with either wild type or PPARα^−/−^ splenocytes had increased cytokine expression, specifically IFNγ and IL4 (Fig. 7d). Together, these data demonstrate the capacity of PPARα^−/−^ splenocytes to reconstitute ConA liver injury and cytokine production to a level equal to or greater than wild type splenocytes. Further, these data suggest that deficiency in PPARα outside of the NKT population (i.e. Hepatocytes, Kupffer cells) are likely responsible for the reductions in NKT cell numbers in PPARα^−/−^ livers.

### PPARα-deficiency does not affect lipopolysaccharide-induced liver injury

**Fig. 8:**
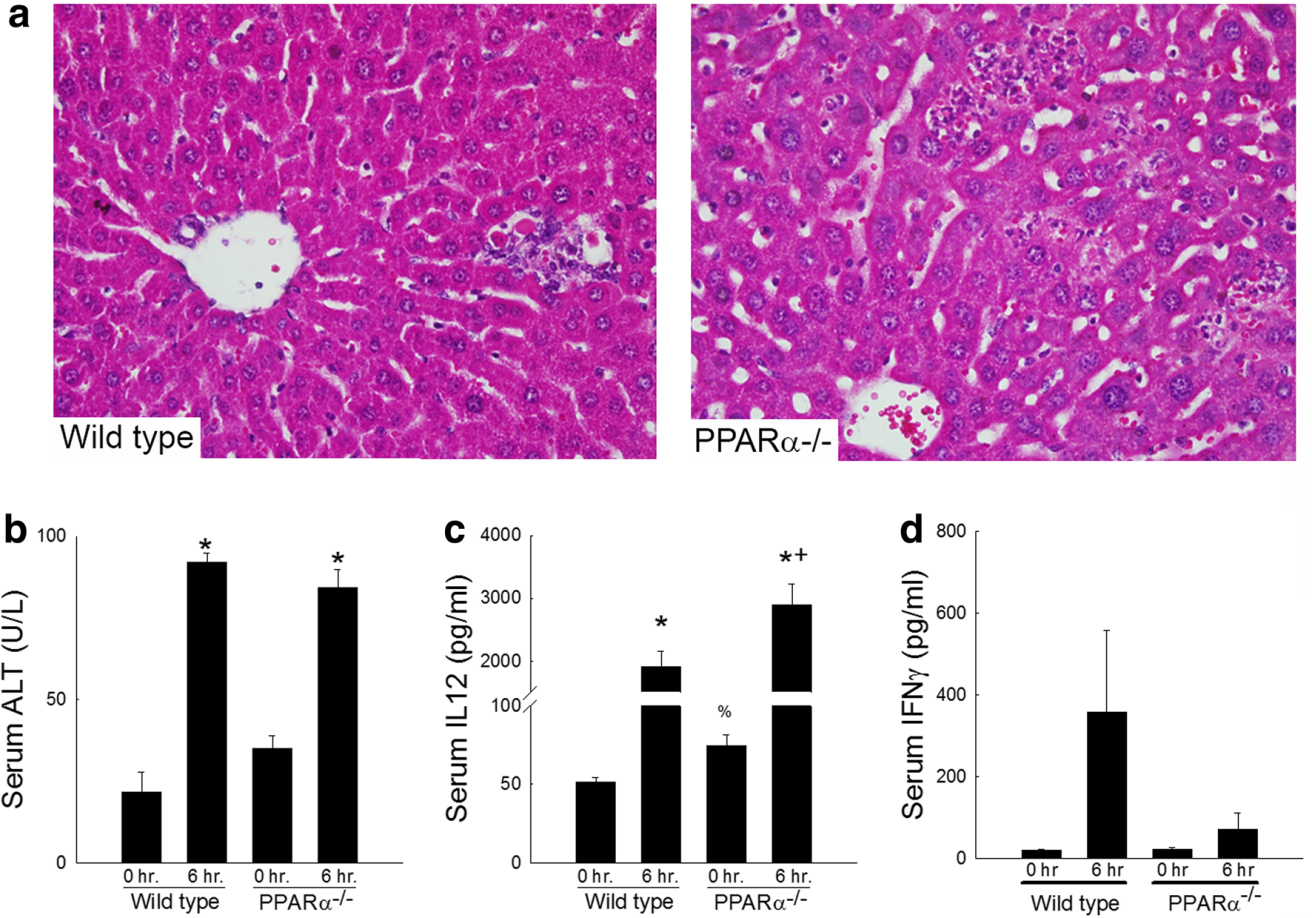
Effect of loss of PPARα on lipopolysaccharide (LPS)-induced liver injury and cytokine response. Wild type and PPARα^−/−^ mice were administered LPS by intraperitoneal injection 6 h prior to sacrifice. **a** Representative photomicrographs of liver sections from wild type or PPARα^−/−^ mice shown at ×400 magnification. **b** Serum alanine aminotransferase (ALT) levels 6 h following LPS exposure. **c** Serum IL12 and **d** serum IFNγ protein levels 6 h following LPS exposure as assessed by ELISA. *p < 0.05 vs. respective vehicle treated control. ^+^p<0.05 vs. LPS-treated wild type mouse

Emerging evidence highlights the involvement of multiple cell populations during ConA-induced liver injury [38]. Specifically hepatic macrophages have been shown to contribute, at least in part, to pro-inflammatory cytokine expression and clotting factor production associated with tissue inflammation and necrosis [39]. Within the current paradigm, reduced NKT cell numbers and reduced IFNγ production correlate with reduced tissue injury in PPARα-deficient mice. The effect which PPARα-deficiency has on macrophage function within the liver has not been thoroughly investigated. To test their responsiveness, wild type and PPARα-deficient mice were administered lipopolysaccharide (5 mg/kg) by intraperitoneal injection 6 h prior to sacrifice. Serum and tissue were collected to assess liver damage and cytokine production. As shown in Fig. 8, LPS administration increased inflammatory cell infiltration as assessed by histopathology, liver injury as measured by serum ALT levels, and significantly increased serum IL12 levels as measured by ELISA. Loss of PPARα did not affect LPS-induced inflammatory cell infiltration or tissue injury but did lead to enhanced serum IL12 levels but reduced serum IFNγ protein. Together, these data suggest that hepatic macrophage function is similar between wild type and PPARα-deficient mice.

### PPARα-deficiency reduces hepatic IL15 expression

**Fig. 9:**
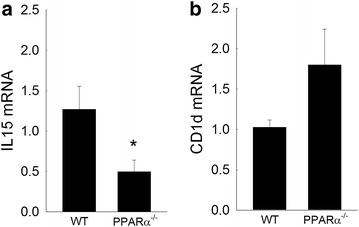
Loss of PPARα reduces hepatic IL15 production but not CD1d expression. Livers of untreated wild type or PPARα^−/−^ mice were examined for the expression of **a** IL15 or **b** CD1d by quantitative PCR. *p < 0.05 vs. wild type control. n = 4–6 mice per group

NKT cell survival is dependent on the expression of key proteins and cell surface molecules [1, 4, 40]. Both hepatic CD1d expression and IL15 production within the liver have been associated with NKT cell survival. Loss of PPARα leads to a reduction in the number of hepatic NKT but the mechanism governing this effect remains unclear. To begin to understand the potential regulators of this response, RNA harvested from untreated wild type or PPARα-deficient mice were examined for the expression of both CD1d and IL15. As shown in Fig. 9, PPARα-deficient livers show reduced expression of IL15 but not CD1d. These data may provide the first mechanistic link between PPARα-deficiency and the absence of hepatic NKT cells.

## Discussion

T cell-dependent liver injury represents an important component of a number of liver pathologies including autoimmune and viral hepatitis [41–43]. Defining the mechanisms by which lymphocytes exert their damaging effects represents an important area of scientific investigation. To this end, the current series of studies have identified PPARα as a potential regulator of hepatic T cells. Specifically, data here demonstrate the importance of PPARα in the recruitment and/or survival of NKT cells, independent of its function within these cells. The ability of PPARα to regulate the immune cell composition of the liver and lymphocyte responses may have important clinical implications in the treatment of a number of T cell-dependent liver pathologies.

ConA-mediated liver injury is a well-described model of T cell dependent acute hepatitis in rodents [16]. NKT cells are activated by ConA in a CD1d-dependent manner to produce IFNγ and IL4 which serve to further activate this cell population as well as recruit and activate additional inflammatory cells including macrophages, thereby acting as a bridge between the innate and adaptive immune response [17–19, 24, 44, 45]. Recent studies by Li et al. as well as studies from our laboratory have drawn a strong correlation between the presence of hepatocellular lipid, absence or reduction in hepatic NKT cells and the production of a shifted T_h_1-type cytokine response in the liver [2, 46]. The results of the current study suggest that the loss of PPARα leads to a similar depletion of hepatic NKT cells which likely contributes to the reduced hepatocellular injury observed following both ConA administration as well as αGal treatment. Importantly, the reduced responsiveness to αGal supports the flow cytometric data indicating reduced NKT cells as numerous reports have shown a potential downregulation of defining cell surface markers, particularly NK1.1 and/or CD49b. Together, these data highlight the deficiency in NKT cells, both in phenotypic appearance and functionality in PPARα deficient mice, a key regulatory immune cell within the normal liver but stop short of defining the mediators of this hepatic immune phenotype.

Hepatic NKT cells are regulated by a variety of factors, both membrane bound as well as secreted. Loss of CD1d, reduced production of supportive cytokines such as IL15 or over-production of inflammatory mediators have all been associated with their depletion [45]. Likewise, activation itself may reduce NKT cell function, alter their cell surface phenotype, or induce cell death. Multiple models of fatty liver have shown interactions with many of these factors. Leptin-deficient ob/ob mice have reduced NKT cell numbers which correlates with reduced hepatic CD1d expression as well as blunted IL15 production [40, 47]. Loss of PPARα did lead to a small but significant reduction in tissue IL15 expression but had no effect on CD1d tissue expression suggesting that PPARα-deficiency, or the accumulation of hepatic lipid that occurs as a result, may influence hepatic production of this important supportive signal as has been noted in other models of fatty liver disease [1, 4, 40]. Choline-deficient diet feeding leads to a time-dependent increase in lipid accumulation and hepatic macrophage IL12 production which inversely correlates with NKT cell numbers [2]. Moreover, genetic deletion of IL12p40 restores the hepatic NKT cell population independent of changes in hepatosteatosis. Within the current study, loss of PPARα leads to a mild microvesicular lipid deposition which correlates with a small but significant increase in serum IL12 production at baseline (Fig. 8). Such data highlight a consistent IL12 response in the presence of excess hepatic lipid accumulation though the mechanism for this upregulation remains unclear. Previous studies reported the ability of PPARα activation to suppress NFκB activation in macrophages limiting their production of a number of inflammatory cytokines [9, 10]. Likewise, loss of PPARα interrupts normal lipid and cholesterol metabolism in macrophages similar to that seen in hepatocytes [48]. Altered lipid homeostasis can have a profound effect on macrophage function, promoting inflammatory cytokine production. Loss of fatty acid binding protein 5 (FABP5) promotes LPS-induced IL12 production in vitro and in vivo from hepatic macrophages further supporting an interaction among lipid, macrophages, and their production of IL12 [3]. The link between IL12 and PPARα at the level of the macrophage remains unclear but is likely related to lipid accumulation and subsequent inflammatory transcription factor activation.

The current series of studies are limited by the global loss of PPARα. Adoptive transfer experiments of lymphocyte populations allow for more selective examination of the effects of this transcription factor. Data from this approach support the notion that loss of PPARα leads to a hepatic microenvironment which is not conducive to NKT cell survival. Supporting this idea, reconstitution of PPARα sufficient, lymphocyte deficient SCID mice with either wild type or PPARα deficient lymphocytes restored ConA-induced tissue injury and cytokine production in these mice. In fact, reconstitution of SCID mice with PPARα-deficient lymphocytes caused a 4 fold enhancement in liver injury when compared to wild type lymphocyte reconstitution. The reasons for this enhancement in tissue injury are not clear. Previous studies have demonstrated the impact of PPARα deficiency on lymphocyte responsiveness [14, 15]. Loss of PPARα exaggerated IFNγ production by CD4^+^ T cells in vitro upon stimulation with CD3 and CD28. Pilot studies confirmed this enhancement in IFNγ production by PPARα deficient lymphocytes (data not shown). In vivo examination of IFNγ production did not, however, reveal significant increases in PPARα reconstituted mice when compared to wild type mice though IL4 levels were doubled. Further examination of the time-course of IFNγ expression is warranted in this setting to better understand its role though data from this study support a function for PPARα independent of the lymphocyte in the regulation of NKT cell function and ConA responsiveness.

Interestingly, in the current study, accumulation of lipid reduces NKT cell numbers and function but does not promote an enhanced Th1 response. This is in contrast to previous studies but may be related to the degree of lipid accumulation as well as the extent of NKT cell depletion. Indeed, previous studies have shown significantly higher levels of lipid accumulation as compared to the current results while also showing significantly higher numbers of hepatic NKT cells remaining following lipid accumulation [37, 40]. It may also be that PPARα regulates the function of other cells with respect to their ability to produce Th1-type cytokines. Data presented in Fig. 8 highlight the ability PPARα-deficiency to enhance lipopolysaccharide-induced IL12 production likely from macrophages but interestingly impair hepatic production of IFNγ. It is clear that macrophages contribute to ConA-induced liver injury as their depletion reduced hepatocellular injury in part through reductions in pro-inflammatory cytokine expression [49]. The involvement of macrophages in the current paradigm remains unclear and reduced IFNγ production following ConA exposure may result from impaired Kupffer cell responses. In vitro studies and αGal administration support a defective NKT cell response but further study is needed to determine the specific source(s) of Th1 cytokines in this and other models and the relative contribution of these cells to overall ConA-induced liver injury. It is clear that loss of PPARα leads to a significant reduction in hepatic NKT cell number and function and limits ConA-induced and αGal stimulated cytokine responses and associated tissue injury.

As discussed above, activation of PPARα stimulates peroxisome proliferation and transcription of a number of lipid metabolizing enzymes in rodents [7]. In humans, PPARα is present at low levels within the liver and does not appear to transactivate genes involved in peroxisomal β oxidation [50]. As such, chronic treatment with PPARα activators does not activate peroxisome or hepatocellular proliferation in humans as it does in rodents. Recent studies have demonstrated that activation of PPARα in human T lymphocytes results in strong reductions in the activation-induced expression of a number of cytokines including IFNγ, a finding consistent with its overall anti-inflammatory effects and its function in this immune cell population [51]. The role that PPARα plays in specific lymphocyte subpopulations as well as in tissue specific localization of these lymphocyte populations in humans has not, however, been explored. Given the results of the present study, modulation of PPARα function within the liver may indirectly modulate the immune response in humans. Additional investigation will be required to determine how PPARα affects lymphocyte function within the human liver.

## Conclusions

In conclusion, data derived from the current series of studies demonstrates the importance of PPARα in the recruitment and/or survival of NKT cells within the liver. Consistent with these reductions in NKT cells, PPARα^−/−^ mice shown strong resistance to ConA-activated and αGal stimulated cytokine production, specifically IFNγ, and subsequent liver damage. The role that other cell populations play in this process, particularly macrophages, cannot be fully addressed in the current paradigm. Further study is required to determine the exact mechanism by which PPARα regulates the localization and/or survival of NKT cells to the liver including the absolute importance of IL15 in this process and the direct contribution of macrophages both in NKT cell survival and tissue injury following ConA exposure. Understanding the mechanisms involved in PPARα-dependent regulation of hepatic immune cell populations may prove useful in the design of therapies to modulate the immunological response of the liver.

## Abbreviations

PPARα: peroxisome proliferator activated receptor alpha
ConA: Concanavalin A
IFNγ: interferon gamma
IL: interleukin
NKT: natural killer T cell
WT: wild type
AOX: acyl CoA oxidase
NFκB: nuclear factor kappa B
CD: cluster determinant
αGal: alpha galactosylceramide
ALT: alanine transaminase
AST: aspartate transaminase
DNA: deoxyribonucleic acid
TUNEL: terminal UTP nick end labeling
ELISA: enzyme linked immunosorbent assay
TCR: T cell receptor
SCID: severe combined immunodeficient
PBS: phosphate buffered saline
T-bet: T box transcription factor expressed in T cells
Th: T helper
FABP: fatty acid binding protein
LPS: lipopolysaccharide

## References

[1] Guebre-Xabier M, Yang S, Lin HZ, Schwenk R, Krzych U, Diehl AM (2000). Altered hepatic lymphocyte subpopulations in obesity-related murine fatty livers: potential mechanism for sensitization to liver damage. Hepatology.

[2] Kremer M, Thomas E, Milton RJ, Perry AW, van Rooijen N, Wheeler MD, Zacks S, Fried M, Rippe RA, Hines IN (2010). Kupffer cell and interleukin-12-dependent loss of natural killer T cells in hepatosteatosis. Hepatology.

[3] Moore SM, Holt VV, Malpass LR, Hines IN, Wheeler MD (2015). Fatty acid-binding protein 5 limits the anti-inflammatory response in murine macrophages. Mol Immunol.

[4] Li Z, Soloski MJ, Diehl AM (2005). Dietary factors alter hepatic innate immune system in mice with nonalcoholic fatty liver disease. Hepatology.

[5] Gonzalez FJ (2002). The peroxisome proliferator-activated receptor alpha (PPARalpha): role in hepatocarcinogenesis. Mol Cell Endocrinol.

[6] Akiyama TE, Nicol CJ, Fievet C, Staels B, Ward JM, Auwerx J, Lee SS, Gonzalez FJ, Peters JM (2001). Peroxisome proliferator-activated receptor-alpha regulates lipid homeostasis, but is not associated with obesity: studies with congenic mouse lines. J Biol Chem.

[7] You M, Crabb DW (2004). Recent advances in alcoholic liver disease II. Minireview: molecular mechanisms of alcoholic fatty liver. Am J Physiol Gastrointest Liver Physiol.

[8] Lovett-Racke AE, Hussain RZ, Northrop S, Choy J, Rocchini A, Matthes L, Chavis JA, Diab A, Drew PD, Racke MK (2004). Peroxisome proliferator-activated receptor alpha agonists as therapy for autoimmune disease. J Immunol.

[9] Marx N, Mackman N, Schonbeck U, Yilmaz N, Hombach V, Libby P, Plutzky J (2001). PPARalpha activators inhibit tissue factor expression and activity in human monocytes. Circulation.

[10] Neve BP, Corseaux D, Chinetti G, Zawadzki C, Fruchart JC, Duriez P, Staels B, Jude B (2001). PPARalpha agonists inhibit tissue factor expression in human monocytes and macrophages. Circulation.

[11] Hennuyer N, Tailleux A, Torpier G, Mezdour H, Fruchart JC, Staels B, Fievet C (2005). PPARalpha, but not PPARgamma, activators decrease macrophage-laden atherosclerotic lesions in a nondiabetic mouse model of mixed dyslipidemia. Arterioscler Thromb Vasc Biol.

[12] Chinetti G, Griglio S, Antonucci M, Torra IP, Delerive P, Majd Z, Fruchart JC, Chapman J, Najib J, Staels B (1998). Activation of proliferator-activated receptors alpha and gamma induces apoptosis of human monocyte-derived macrophages. J Biol Chem.

[13] Mishra A, Chaudhary A, Sethi S (2004). Oxidized omega-3 fatty acids inhibit NF-kappaB activation via a PPARalpha-dependent pathway. Arterioscler Thromb Vasc Biol.

[14] Jones DC, Ding X, Daynes RA (2002). Nuclear receptor peroxisome proliferator-activated receptor alpha (PPARalpha) is expressed in resting murine lymphocytes. The PPARalpha in T and B lymphocytes is both transactivation and transrepression competent. J Biol Chem.

[15] Jones DC, Ding X, Zhang TY, Daynes RA (2003). Peroxisome proliferator-activated receptor alpha negatively regulates T-bet transcription through suppression of p38 mitogen-activated protein kinase activation. J Immunol.

[16] Tiegs G, Hentschel J, Wendel A (1992). A T cell-dependent experimental liver injury in mice inducible by concanavalin A. J Clin Invest.

[17] Takeda K, Hayakawa Y, Van Kaer L, Matsuda H, Yagita H, Okumura K (2000). Critical contribution of liver natural killer T cells to a murine model of hepatitis. Proc Natl Acad Sci USA.

[18] Jaruga B, Hong F, Sun R, Radaeva S, Gao B (2003). Crucial role of IL-4/STAT6 in T cell-mediated hepatitis: up-regulating eotaxins and IL-5 and recruiting leukocytes. J Immunol.

[19] Jaruga B, Hong F, Kim WH, Gao B (2004). IFN-gamma/STAT1 acts as a proinflammatory signal in T cell-mediated hepatitis via induction of multiple chemokines and adhesion molecules: a critical role of IRF-1. Am J Physiol Gastrointest Liver Physiol.

[20] Lee SS, Pineau T, Drago J, Lee EJ, Owens JW, Kroetz DL, Fernandez-Salguero PM, Westphal H, Gonzalez FJ (1995). Targeted disruption of the alpha isoform of the peroxisome proliferator-activated receptor gene in mice results in abolishment of the pleiotropic effects of peroxisome proliferators. Mol Cell Biol.

[21] Kremer M, Perry AW, Milton RJ, Rippe RA, Wheeler MD, Hines IN (2008). Pivotal role of Smad3 in a mouse model of T cell-mediated hepatitis. Hepatology.

[22] Hines IN, Kremer M, Isayama F, Perry AW, Milton RJ, Black AL, Byrd CL, Wheeler MD (2007). Impaired liver regeneration and increased oval cell numbers following T cell-mediated hepatitis. Hepatology.

[23] Peters JM, Rusyn I, Rose ML, Gonzalez FJ, Thurman RG (2000). Peroxisome proliferator-activated receptor alpha is restricted to hepatic parenchymal cells, not Kupffer cells: implications for the mechanism of action of peroxisome proliferators in hepatocarcinogenesis. Carcinogenesis.

[24] Toyabe S, Seki S, Iiai T, Takeda K, Shirai K, Watanabe H, Hiraide H, Uchiyama M, Abo T (1997). Requirement of IL-4 and liver NK1+ T cells for concanavalin A-induced hepatic injury in mice. J Immunol.

[25] Tagawa Y, Kakuta S, Iwakura Y (1998). Involvement of Fas/Fas ligand system-mediated apoptosis in the development of concanavalin A-induced hepatitis. Eur J Immunol.

[26] Zhang H, Cook J, Nickel J, Yu R, Stecker K, Myers K, Dean NM (2000). Reduction of liver Fas expression by an antisense oligonucleotide protects mice from fulminant hepatitis. Nat Biotechnol.

[27] Seino K, Kayagaki N, Takeda K, Fukao K, Okumura K, Yagita H (1997). Contribution of Fas ligand to T cell-mediated hepatic injury in mice. Gastroenterology.

[28] Marzio R, Mauel J, Betz-Corradin S (1999). CD69 and regulation of the immune function. Immunopharmacol Immunotoxicol.

[29] Radaeva S, Sun R, Pan HN, Hong F, Gao B (2004). Interleukin 22 (IL-22) plays a protective role in T cell-mediated murine hepatitis: IL-22 is a survival factor for hepatocytes via STAT3 activation. Hepatology.

[30] Sun R, Tian Z, Kulkarni S, Gao B (2004). IL-6 prevents T cell-mediated hepatitis via inhibition of NKT cells in CD4^+^ T. J Immunol.

[31] O’Garra A, Arai N (2000). The molecular basis of T helper 1 and T helper 2 cell differentiation. Trends Cell Biol.

[32] Agnello D, Lankford CS, Bream J, Morinobu A, Gadina M, O’Shea JJ, Frucht DM (2003). Cytokines and transcription factors that regulate T helper cell differentiation: new players and new insights. J Clin Immunol.

[33] Grogan JL, Locksley RM (2002). T helper cell differentiation: on again, off again. Curr Opin Immunol.

[34] Underhill GH, Zisoulis DG, Kolli KP, Ellies LG, Marth JD, Kansas GS (2005). A crucial role for T-bet in selectin ligand expression in T helper 1 (Th1) cells. Blood.

[35] Siebler J, Wirtz S, Klein S, Protschka M, Blessing M, Galle PR, Neurath MF (2003). A key pathogenic role for the STAT1/T-bet signaling pathway in T-cell-mediated liver inflammation. Hepatology.

[36] Ishihara S, Nieda M, Kitayama J, Osada T, Yabe T, Kikuchi A, Koezuka Y, Porcelli SA, Tadokoro K, Nagawa H, Juji T (2000). Alpha-glycosylceramides enhance the antitumor cytotoxicity of hepatic lymphocytes obtained from cancer patients by activating CD3–CD56+ NK cells in vitro. J Immunol.

[37] Kremer M, Hines IN, Milton RJ, Wheeler MD (2006). Favored T helper 1 response in a mouse model of hepatosteatosis is associated with enhanced T cell-mediated hepatitis. Hepatology.

[38] Wang HX, Liu M, Weng SY, Li JJ, Xie C, He HL, Guan W, Yuan YS, Gao J (2012). Immune mechanisms of Concanavalin A model of autoimmune hepatitis. World J Gastroenterol.

[39] Ju C, Tacke F (2016). Hepatic macrophages in homeostasis and liver diseases: from pathogenesis to novel therapeutic strategies. Cell Mol Immunol.

[40] Li Z, Lin H, Yang S, Diehl AM (2002). Murine leptin deficiency alters Kupffer cell production of cytokines that regulate the innate immune system. Gastroenterology.

[41] Haydon G, Lalor PF, Hubscher SG, Adams DH (2002). Lymphocyte recruitment to the liver in alcoholic liver disease. Alcohol.

[42] Bowen DG, Walker CM (2005). Adaptive immune responses in acute and chronic hepatitis C virus infection. Nature.

[43] Ichiki Y, Aoki CA, Bowlus CL, Shimoda S, Ishibashi H, Gershwin ME (2005). T cell immunity in autoimmune hepatitis. Autoimmun Rev.

[44] Godfrey DI, MacDonald HR, Kronenberg M, Smyth MJ, Van Kaer L (2004). NKT cells: what’s in a name?. Nat Rev Immunol.

[45] Exley MA, Koziel MJ (2004). To be or not to be NKT: natural killer T cells in the liver. Hepatology.

[46] Li Z, Diehl AM (2003). Innate immunity in the liver. Curr Opin Gastroenterol.

[47] Hua J, Ma X, Webb T, Potter JJ, Oelke M, Li Z (2010). Dietary fatty acids modulate antigen presentation to hepatic NKT cells in nonalcoholic fatty liver disease. J Lipid Res.

[48] Rigamonti E, Chinetti-Gbaguidi G, Staels B (2008). Regulation of macrophage functions by PPAR-alpha, PPAR-gamma, and LXRs in mice and men. Arterioscler Thromb Vasc Biol.

[49] Schumann J, Wolf D, Pahl A, Brune K, Papadopoulos T, van Rooijen N, Tiegs G (2000). Importance of Kupffer cells for T-cell-dependent liver injury in mice. Am J Pathol.

[50] Palmer CN, Hsu MH, Griffin KJ, Raucy JL, Johnson EF (1998). Peroxisome proliferator activated receptor-alpha expression in human liver. Mol Pharmacol.

[51] Marx N, Kehrle B, Kohlhammer K, Grub M, Koenig W, Hombach V, Libby P, Plutzky J (2002). PPAR activators as antiinflammatory mediators in human T lymphocytes: implications for atherosclerosis and transplantation-associated arteriosclerosis. Circ Res.

